# Community resistance to a peer education programme in Zimbabwe

**DOI:** 10.1186/s12913-014-0574-5

**Published:** 2014-11-19

**Authors:** Catherine Campbell, Kerry Scott, Zivai Mupambireyi, Mercy Nhamo, Constance Nyamukapa, Morten Skovdal, Simon Gregson

**Affiliations:** Department of Social Psychology, London School of Economics and Political Science, Houghton Street, WC2A 2AE London, UK; Department of International Health, Johns Hopkins Bloomberg School of Public Health, Baltimore, MD USA; Biomedical Research and Training Institute, Harare, Zimbabwe; School of Public Health, Imperial College, London, UK; Department of Public Health, University of Copenhagen, Copenhagen, Denmark

**Keywords:** HIV/AIDS intervention, Programme evaluation, Africa, Community mobilisation, Commercial sex work

## Abstract

**Background:**

This paper presents community perceptions of a state-of-the-art peer education programme in Manicaland, Zimbabwe. While the intervention succeeded in increasing HIV knowledge among men and condom acceptability among women, and reduced HIV incidence and rates of unprotected sex among men who attended education events, it did not succeed in reducing population-level HIV incidence. To understand the possible reasons for this disappointing result, we conducted a qualitative study of local perspectives of the intervention.

**Methods:**

Eight focus group discussions and 11 interviews with 81 community members and local project staff were conducted. Transcripts were interrogated and analysed thematically.

**Results:**

We identified three factors that may have contributed to the programme’s disappointing outcomes: (1) difficulties of implementing all elements of the programme, particularly the proposed income generation component in the wider context of economic strain; (2) a moralistic approach to commercial sex work by programme staff; and (3) limitations in the programme’s ability to engage with social realities facing community members.

**Conclusions:**

We conclude that externally-imposed programmes that present new information without adequately engaging with local realities and constraints on action can be met by resistance to change.

## Background

This paper provides a detailed case study of community and local health worker perceptions of a, at the time, state-of-the-art peer education HIV prevention programme in Zimbabwe. It draws attention to how a poor fit between global expectations and local realities undermined the outcome of the intervention.

Most of the 34 million people living with HIV and AIDS globally live in sub-Saharan Africa [1]. HIV continues to spread in the region, outpacing efforts to scale up antiretroviral therapy, often with devastating impact on family and community life. In 2010, 70% of all new HIV infections (1.9 million people) were found in sub-Saharan Africa [1]. Heterosexual intercourse remains the epidemic’s driving force in sub-Saharan Africa and commercial sex work plays a major role [2]. Zimbabwe’s adult prevalence rate amongst adults between 15 and 49 was 14.7% at most recent measure [3], making it one of the hardest hit countries in sub-Saharan Africa and the world.

In response, billions of dollars have been poured into interventions that have sought to reduce the spread of HIV. Declines in HIV infection rates, while heartening, have not been conclusively linked to behaviour change interventions rather than the natural course of the epidemic or behaviour changes brought about through indigenous or individual responses [4,5]. Randomized control trials examining the impact of behaviour change interventions have found them to have had no overall impact on HIV incidence rates [6-8]. A similar pattern has been observed in peer education programmes for HIV prevention [9]. Reviewing thirty studies evaluating the impact of peer education programmess in developing country contexts, Medley et al. [9] found that peer education has no significant effect on sexually transmitted infections and is only moderately effective at improving behavioural outcomes. Medley et al. [9] call for research that can explain these disappointing results and be used as a springboard to improve the impact of peer education programmes. Reporting on the experiences of peer education programmes in South Africa, Campbell and colleagues highlight that much remains to be learned about the complexities of translating theoretically sound social development for health programmes, such as peer education for HIV prevention, into practice [10-12].

Elsewhere, we have argued that peer education is a method with excellent potential, rooted in sound social psychological understandings of the capacity for collective action to facilitate the renegotiation of health-damaging social norms [13]. In the face of disappointing programme outcomes, we have suggested that greater attention needs to be paid to the following factors if the method is to reach its full potential: (i) whether peer education programmes are implemented in ways that are truly ‘dialogical’ and ‘bottom up’, connecting with the lifeworlds of their target audience (rather than being designed and imposed on target communities by outside health agencies who fail to understand local realities); and (ii) whether appropriate contextual supports are put in place to facilitate programme effectiveness. To highlight the social dynamics that may influence peer education outcomes, this paper explores local community perceptions of the peer education component of an STI/HIV intervention in rural Zimbabwe. By way of introduction, we will first discuss the theory underlying our research and introduce the intervention and the study.

### Theoretical contribution: rethinking evaluation research

Taking up the concern expressed by development anthropologist David Mosse [14], we seek to move away from evaluation approaches that place the onus of success or failure solely on the beliefs and behaviours of target communities, with disappointing results explained by blaming factors such as local culture, lack of support by local political leaders or technical minutiae of programme messages. Instead we seek to contextualise the programme more widely, focusing on the extent to which the intervention was able to generate social environments that supported the possibility of health-enhancing behaviour change.

When interventions succeed or fail it is vital to examine why they have done so to inform future policy and practice [15]. Our theoretical approach centres on Pawson and Tilley’s [16] concept of ‘realistic evaluation’ that advised programme evaluators to pay attention both to features of the programme being implemented and the context in which the programme takes place in order to understand outcomes. Pawson and Tilley [16] are skeptical of traditional evaluation methods that ask “Does this work?” or “What works?” Instead, they encourage researchers to ask “What works for whom in what circumstances?” because of the highly different effects of interventions on different people and in different contexts. This evaluation approach fits into the ‘enabling contexts’ approach to health promotion introduced by Tawil, Verster and O’Reilly [17]. This approach understands health behaviour not simply as the result of individual behavioural decisions but also as responsive to the contexts in which people live. It advocates that health interventions try not only to persuade people to change their behaviour, but also to seek to reframe the environmental contexts in which people make choices, examining the extent to which interventions enable people to change through building better environments [18].

There has been a tendency for programme evaluators to blame unhelpful beliefs or behaviours of target community members as their key explanatory variables, with insufficient attention paid to how the designs take account of and fit with the local social context. Evaluations of disappointing programmes too often focus on events, situations and people outside of the framework of health or development expertise and authority when trying to determine what went wrong [14]. They may cite problematic local culture or gender attitudes that led local people to resist the intervention. Within the field of HIV prevention, there is growing emphasis on the need for evaluators to take account of contextual factors, particularly how the intervention is implemented, its relevance to the community [19] and the power dynamic between local actors, international donors and the intervention team [20,21].

### The Manicaland STI/HIV Intervention

A team of researchers from the UK and Zimbabwe conducted a cluster-randomized control trial that investigated the effectiveness of combined community- and clinic-based HIV prevention in Manicaland, eastern Zimbabwe. At the time of the trial, the HIV prevalence in Manicaland was around 20% [22]. A detailed description of the STI/HIV intervention, data collection, analysis and results is presented in Gregson, et al*.* [23]. Here we provide an overview of the Manicaland STI/HIV Intervention, which this present paper explores through qualitative follow-up interviews and focus groups.

Between 1998 and 2003, six communities in Manicaland received targeted and population-level programmes to reduce HIV incidence by (a) promoting safer sexual behaviour and (b) improving the treatment of STIs that facilitate HIV-1 transmission. Safer sexual behaviour was defined as later sexual debut, fewer non-regular partnerships in the past month and less unprotected sex with regular and casual partners in the past three years. Six randomly selected pair communities did not receive the intervention but continued to receive standard Government healthcare services, including basic STI management, social marketing of male and female condoms, condom distribution, and limited HIV/AIDS–focussed awareness meetings and poster/flyer campaigns. The intervention was to consist of (1) peer education and condom distribution amongst commercial sex workers (CSWs) and male clients at workplaces, beer halls and in the general community, supported by income-generating projects; (2) strengthened STI services at local health centres; and (3) open days with HIV/AIDS education activities at health centres to promote safer sexual behaviour and to increase the uptake of local STI treatment services. The specific objectives of the peer education component were to i) recruit, train and motivate peer educators to reach the wider community and sexually vulnerable groups; ii) develop an outreach programme among CSWs and clients; iii) develop single women’s associations and networks. The peer educators were selected from local single women (mainly divorced, separated or widowed), most of whom were CSWs (with a mix of past or active CSWs), representing all age groups. The programme rationale was that such women would be supported in reducing unprotected sex and in leaving commercial sex work through income generating projects. A total of about 63,000 peer-education meetings were held, covering a schedule with a variety of activities such as brief presentations about HIV, discussions, drama, role plays and videos on safe sexual behaviour, condom demonstrations and quizzes. A total of 6.8 million condoms were distributed by the programme in the intervention communities.

The overall results of the cluster-randomised controlled trial (N = 5228) conducted to measure the impact of the CSW intervention were disappointing [23]: whilst, HIV incidence and unprotected sex were reduced amongst men who attended peer education meetings in the intervention communities, no reductions in HIV incidence or in associated risk behaviours was found between the intervention communities and control communities.

## Methods

The scope and limitations of this paper are re-emphasised here: First, we do not seek to comment on the wider integrated programme as a whole but only on the peer education component. Second, in commenting on the peer education programme, we do not seek to provide any definitive ‘diagnosis’ of programme strengths and weaknesses, but only to report – in a more limited way – on community perceptions of these factors. We emphasise here that community views present a partial lens on what happened, and that there will be other perspectives (from epidemiology, behavioural science and biomedicine for example), which might offer other explanations. Having said that, we believe the perceptions of members of the programme’s target audiences provide one valuable piece in the wider puzzle of explaining the outcomes of a complex programme implemented in a complex social setting.

Against this background, this paper presents qualitative follow-up research into the experiences and opinions of community members and those closely associated with the intervention on the ground. Over a two-week period in 2006, we spoke to 81 people through eight focus group discussions (FGDs) and 11 individual interviews. We held the following FGDs: one with peer educators (who were also current or former CSWs); one with CSWs who were not peer educators; three with men who frequent CSWs, and three with general community members. Our individual interviews were with: two peer educators (current or former CSWs), five staff members from local clinics in the intervention communities that offered improved STI and HIV services, and two project implementers. Table 1 provides a detailed description of our dataset.

**Table 1:** **Dataset**

**Type**		**Informant type**	**Participants**
FGDs	1	Peer educators/CSWs	8 females
	2	CSWs	9 females
	3	Male clients	8 males; clients of CSWs
	4	Male clients	6 males; clients of CSWs
	5	Male clients	12 males; clients of CSWs
	6	General public	7 participants, male & female
	7	General public	10 participants, male & female
	8	General public	10 females
Interviews	1	Peer educator/CSW	Female
	2	Peer educator	Male
	3	Key informant (clinic staff)	Male
	4	Key informant (clinic staff)	Male
	5	Key informant (clinic staff)	Male
	6	Key informant (clinic staff)	Male
	7	Key informant (clinic staff)	Male
	8	Nurse (Sister-in-Charge)	Female
	9	Nurse (Nurse-in-Charge)	Male
	10	Project implementer (Project Co-ordinator)	Female
	11	Project implementer (Peer education co-ordinator)	Female
**Total**		**Total: 8 FGDs & 11 interviews**	**81 people**

Participants were recruited through convenience sampling at a random selection of intervention sites. The FGDs and interviews took place over a two-week period. All people approached to participate agreed. Informed and written consent was obtained from all participants with the agreement that their confidentiality would be ensured. FGD participants were given a large block of soap and interviewees were given a T-shirt to compensate them for their time. Prior ethical approval was obtained from the Research Council of Zimbabwe – Number 02187 – the Applied and Quality Research Ethics Committee in Oxford, United Kingdom – N97.039 – and UNAIDS Research Ethics Committee – ERC 98/03.

Questions varied depending on the FGD participants or interviewee but generally included: whether and how they had experienced elements of the intervention, particularly the peer education component; whether they had learned anything; what they thought about the model of having current or former CSWs become peer educators; how people cope with STIs; local thoughts on condom use; and whether they had seen any behaviour change. Participants/interviewees were also asked “We distributed a million condoms in this community, but there are just a few that were used; what do you think happened to the rest of them?” “STI/HIV rates were reported to be the same or higher in the intervention sites as the control sites. What do you think could have caused this?” and, specifically for the project implementers “Were there implementation problems? What were they?”

The FGDs and interviews took between an hour and an hour and a half. They were conducted in Shona by the third and fourth authors, both social workers and experienced qualitative fieldworkers, and recorded then translated and transcribed into English.

### Analysis

We used Attride-Sterling’s thematic network analysis technique [24] to analyse the transcripts. This analysis first involved reading and rereading the data. Second, we went through the transcripts and summarized text segments (usually one to five sentences long) categorised according to their basic theme. The basic theme is the underlying idea that a text segment expresses, such as “sex workers must reform” or “sex workers are not respectable”. These basic themes were then grouped into organizing themes and then universal themes, such as “moralistic attitude about sex work”. We developed three universal themes that speak to our research interest of why the STI/HIV intervention had disappointing results and serve as the three sub-headings in the findings section, below.

## Results and discussion

We found widespread awareness of the intervention, with nearly everyone in the focus groups, from male clients of sex workers to married women, expressing some level of familiarity with the programme. The predominant community understanding was that the intervention consisted of distributing condoms and ‘reforming’ commercial sex workers (i.e. getting them to stop selling sex) then training them to become peer educators. The peer education was understood to consist of conducting educational talks and drama skits in the community on HIV, primarily in beer halls (the main site of arranging transactional sex), as well as workplaces and local clinics. The following quotations are a sample of how community members described the intervention:

So what they [peer educators] would do is that usually Monday or Wednesday morning they will go and talk to people at the work place. Maybe you will be a group of about 10. They will talk to you and they will act their drama and after that they will ask questions. Mostly they will be teaching about sexual issues… (General public FGD 6, person 6)

These [peer educators] did educate us. Like myself, I didn’t know how to put on a condom for quite a long time but through their programme which they used to do in the beer hall when they would do a demonstration on how to put on a condom. After that it became easy for me to put on a condom. Because in some cases the condom would just tear off while I was trying to put it on. (Male clients FGD 4, person 1)

The key messages that people reported from the intervention were from the peer educators and focused on how to use a condom (as described in the quotation above) and not being promiscuous:

In such dramas they would depict how sleeping around may result in someone’s death. Their role plays were good because they would make people understand their message of what would likely happen if someone sleeps around. (Male clients FGD 4, person 5)

FGD participants and interviewees frequently expressed conflicted and shifting opinions about the Manicaland STI/HIV Intervention. FGDs and interviews generally began with positive responses, where community members discussed how much they appreciated the intervention and how much they learned, perhaps in part because they perceived the interviewer to be affiliated with the intervention^a^. People in highly resource-poor settings, as Manicaland certainly is, may have an incentive to please outside researchers or programme implementers in order to retain ties to networks that represent a link to funding and potential future interventions.

However, as the interviewer gained rapport and emphasized the need for frankness, participants began discussing the elements of the intervention that they did not like or understand and often revealed that their sexual health-related behaviours and attitudes towards condoms had remained unchanged and resistant to the intervention’s messages of faithfulness and condom use. For instance, at the beginning of interview 1 with a peer educator she reported that “people accept condoms; we have not faced any resistance towards condom use”. Later in the interview she had the following exchange where it became apparent that condoms were not popular and used very irregularly:

**Interviewer:** How many clients do you think CSW have per week?

**Peer educator:** An average of twenty-one clients per week.

**Interviewer:** How many condoms does a single commercial sex worker use per week?

**Peer educator**: Approximately seven condoms per week.

**Interviewer:** Do they use condoms all the time they have sex?

**Peer educator:** No they don’t use condoms all the time, they only use condoms with a new client but as soon as they get used to each other they abandon condom use.

**Interviewer:** What are your views regarding condom use?

**Peer educator:** It’s not feasible to use a condom all the time. Please try and find another safer method if there is any. (Peer educator interview 1)

People expressed conflicted attitudes towards the model of training former CSWs to become peer educators. Many appreciated the idea of getting women who were open about sex and who could freely enter beer halls to become educators. However at the same time, people questioned how the community could respect the views of such women, who were widely disrespected by other community members. They operate in a context where promiscuous sex and alcohol consumption by women was highly problematic. We discuss community reflections on the intervention’s approach of having CSWs become peer educators in more detail in subsection “Lack of meaningful engagement with the social realities in the community”. These inconsistent and conflicted attitudes resonate with Gregson et al*’*s [23] findings from the quantitative analysis of the intervention’s limited and inconsistent outcomes.

The three themes that emerged from our analysis of community perceptions of the intervention’s lack of success in building supportive community contexts for change were as follows: obstacles to actually implementing all elements of the intervention, moralistic approach to commercial sex work that exacerbated stigma and reduced chances for sex worker solidarity, and challenges engaging with the social realities facing community members. We now move on to discussing these themes.

### Challenges in implementing all components of the intervention

The Manicaland STI/HIV Intervention proposal was multifaceted, as discussed earlier. However informants reported that several key elements were not fully implemented, hindering the capacity for other elements of the intervention to succeed.

One programme implementer (PI2, interview 11) listed multiple implementation issues challenging the programme. These included a lack of monitoring and supervision of the peer education programme from the implementing parties; the failure of one of the four key NGO partners to deliver the co-ordination and support promised; and another of the key partners failing to deliver funding pledged to some of the income generating projects.

CSWs trained to be peer educators commented that they did not receive follow-up training and had trouble remembering what they learned during the peer education training, as the following quotation describes:

Refresher courses should be improved at least they should be done within a short duration not after a long time when we have forgotten what we once trained. The other issue is that [a partner NGO] should visit us on a regular basis and furnish us with information, education and communication material. We kindly ask [another partner NGO] to help us also when conducting refresher courses (Peer educator interview 1)

Even in those cases where programme funding was available, the income generating projects were not successful. The programme implementer PI2 explained that the projects were unsustainable because of tough economic conditions, drought and inadequate NGO support: A revolving loan given to a single women’s association (which was to help CSWs in three of the six intervention sites) decreased significantly in value because of inflation^b^. Proposals that seed loans to ‘reformed’ CSWs would be repaid after harvesting did not work, because of poor harvests. Another rotating credit club introduced by the intervention sought to pool members’ resources to invest in small income generating projects and share proceeds among members on a revolving basis. However, it too dissolved when the money lost value because of inflation. Grants and loans for income generating projects given by another NGO were used by former CSWs to buy materials and dyes for a textile project. In that instance, the beneficiaries did not understand that their efforts were supposed to become self-sustaining and expected the NGO to continue to contribute money for more materials and dyes. They then abandoned the project when on-going support was not forthcoming. An income generating project in which former CSWs knitted gloves to sell to people working on forestry estates received a donation of wool but was not economically sustainable.

Without sustainable income generating projects, the economic elements of the local context continued to be those of poverty and unemployment. Sex work remained a viable survival strategy:

[The CSWs] didn’t change their behaviour because they were not given other incentives such as money for survival (key informant interview 7, clinic nurse)

Nonetheless sex workers continued to be exposed to the programme message that they should stop selling sex and earn money in another way. In the following quotation, CSW 1 in FGD 1 says the programme ‘advised’ them to do self-help projects and told them that sex work is not the only way of earning money:

…They [the peer educators who transmitted the program messages] only advised people on starting up self-help projects. They stated that prostitution is not the only way of raising income but to be hardworking doing different projects like fruit vending (CSW FGD 1, person 3).

This message was, for most CSWs, untrue: sex work continued to be the only feasible way to earn money. The message that alternative sources of income are available, if only they were ‘hardworking’ enough, failed to recognize the economic reality facing the sex workers. Considering that the context in which sex workers operated did not change, it is unsurprising that their sexual behaviour remained the same. The next section examines the moralistic elements of the intervention’s message more deeply.

### Moralistic and unrealistic approach to commercial sex work that exacerbated stigma, reduced chances for sex worker solidarity and undermined sex worker credibility

The CSWs who became peer educators felt pressure to leave the sex industry because alternative means of earning income would be made available to them. For example, a CSW recounts that she has been encouraged to start an income generating project and be faithful:

They encouraged us to start income generating projects and to be faithful to one partner in order to reduce the risk of being infected with HIV/AIDS. (CSW 6, FGD, site 7)

From the onset, the attitude of ‘rehabilitating’ CSWs expressed by local project implementers implied that their choice of work was undesirable – perpetuating their stigmatisation. Anti-sex work messages and an emphasis on monogamy was ingrained in the language of both project staff and the CSW peer educators and conveyed through education sessions and skit performances. Participants in FGDs and interviews repeatedly referred to the fact that the peer educators had not ‘reformed’ and speculated about the extent to which they reduced their number of clients. For example, one key staff member commented that:

Some of the peer educators were not honest; the idea was that they would reform once they assume the peer educator’s role but most of the peer educators did not reform. Instead they continued to fight for the same male clients with their peers (PI2, interview 11)

In discussions between intervention staff and community members, staff asked if the peer educators were ‘better now’ (i.e. had stopped selling sex) and whether they served as good examples by influencing non-peer educator CSWs to ‘leave that behaviour’.

Despite the failure of the income generating projects to enable CSWs to move out of sex work, the community members we spoke to reported on-going exposure to messages that CSWs, particularly those selected to be peer educators, should ‘reform’ by leaving sex work and practice and promote monogamy. Unsurprisingly, community members expressed amusement or contempt towards practicing CSWs who spoke against sex work. Community members were aware of the sex workers’ continued source of income from sex work, and felt that the promotion of anti-CSW messages undermined the profile of the project, as this male client notes:

It was easy to learn [the risks of promiscuity] from their dramas but they themselves were not able to practice what they were teaching (Male clients FGD 4, person 4)

Since community members understood the validity of peer educators as contingent on them giving up sex work (something that was not economically possible), community members reported that they could not take the peer educators seriously. This male client of CSWs explains:

After they change they can then educate other people, but, if they don’t change, people will not listen to their words that are not accompanied by the appropriate behaviour (Male clients FGD 5, person 1)

The additional messages that the CSW peer educators delivered (for example, how to correctly use a condom) lacked salience due to a perceived lack of credibility of the deliverers. Moreover, in order to continue their commercial sex work, they needed men to have sex with them, putting them in a further difficult position of simultaneously transmitting messages of faithfulness and needing to recruit clients, discussed in the following:

I once came across a peer educator and she invited me to her place… This did not go down well with me because I thought that she was not supposed to look for sexual clients because she is a peer educator (Male clients FGD 3, person 8)

You know the problem is that these peer educators, after working, they resort back to their old style of being promiscuous. They take advantage of their position to advertise themselves. I have an example of one peer educator who is dating a married man. (Key informant interview 4, clinic staff, MP01)

This incompatible position was noted by many sex workers, clients and key informants and was cited as evidence of the peer educators’ failure, rather than the programme’s problem.

The interviewees and FGD participants emphasized that the programme focused on ending sex work, which did not improve a sense of solidarity or empowerment among CSWs. As the following quotation highlights, sex workers operated in competitive and unfriendly environments, making it difficult for them to discuss sexual health issues and strategies:

From the way I look at it, a prostitute and a prostitute can’t be good friends. And some of the peer educators are prostitutes so the people [CSWs] won’t listen to the message that the person will be saying. They despise both the message and the person (Peer educator/CSW FGD 1, person 9)

Sex workers were thus positioned by those we spoke to (including CSWs themselves) as not ‘hardworking’, ‘lacking in self-control’, seemingly unaware of a multitude of other income generating options and having to give up sex work in order to be capable of educating others. This approach created a very difficult environment for peer educators to operate in and reduced the capacity of male clients and other CSWs to benefit from the educative elements of the intervention.

### Lack of meaningful engagement with the social realities in the community

Our analysis of community perceptions suggests that, from their perspective, the peer education programme design (with its focus on the provision of information and condoms) was a blunt instrument in the complex social context in which people accessed and interpreted information and made sexual behaviour choices. The respondents suggested that the programme failed to adequately take account of their local realities, particularly around the issue of message diffusion and condom use.

Although local people believed that HIV messages were supposed to diffuse from the commercial sex workers to wider society, they commented that this diffusion was not feasible. This lack of diffusion is echoed in Gregson et al*’*s [23] finding that men who were actually exposed first-hand to peer education meetings had reduced rates of STIs and HIV; such reductions were not found in women and the general community who had not attended meetings. The existing ‘segregated’ social system, with high levels of stigma and disapproval of sex work, created an often impenetrable social boundary between sex workers and ‘respectable’ women.

We found that many men socialised with both groups of women, interacting with CSWs whilst also participating in ‘respected’ society either through marriage or eligibility for marriage. In so doing, they were secretive about their contact with sex workers. If their wives were aware of this contact, they often chose to turn a blind eye to it.

This led to a three-fold complexity: first, women sought to distance themselves from CSWs and thus limited their own exposure to the peer education messages. Second, men who were exposed to the peer educators were very unlikely to talk openly about what they learned to their wives, because that would be seen as evidence of infidelity. Third, married women were displeased with peer educators trying to talk to men since they felt that peer educators were still CSWs seeking clients.

Married women maintained a distance from CSWs. This distance was both physical, in that they would not visit the same places as CSWs, and emotional or intellectual, in that they would not value and listen to messages put forth by CSWs. The following quotations highlight the distance between CSWs and other women:

It [the model of training CSWs to be peer educators] was not good. They should have chosen good people who are fit to do that, people who can control their behaviour. (General public FGD 6, female, person 7)

These people [peer educators] did not have the respect of other women. It is very difficult for other women to see anything good coming from prostitutes. So other women did not even value that programme (Male clients FGD 4, person 2)

Non-CSW women avoided beer halls, a key area where much of the intervention took place. Beer halls are the site of a large portion of commercial sex arrangements and are frequented by men and unmarried women who are generally soliciting commercial sex partners. Married, self-described ‘respectable’ women would never go to a beer hall and married men would never want their wives to know they visited one since it is often considered tantamount to having bought sex. If a married man was exposed to the intervention’s messages he would not be able to discuss it at home with his wife because it was seen as evidence that he had interacted with a sex worker. The following quotation highlights this dilemma:

If we get home and try to use condoms with our wives they think that we have been practicing using condoms in beer halls (Male clients FGD 5, person 12)

Beyond distancing themselves from CSWs, married women were also concerned with peer educators trying to speak to their husbands in the community and at work as the following quotation discusses.

I think it was a good approach because it would make the peer educators themselves realize that what they were doing was not good because it was fuelling the spread of HIV. But it did not really work because when wives get to see these commercial sex workers educating their husbands, they would think that these commercial sex workers are now after their husbands, so it also needs respectable people to do such kind of work. (Male clients FGD 4, person 5)

Finding educators and venues where women and men beyond CSWs and clients could be exposed to the intervention’s messages may have been valuable, as is suggested here:

I think it was a good idea to ‘send a thief to catch a thief’ because it was a way of making sure that those who are infected would not continue to infect those who are not infected. But I also think it would be proper to include those who are not ‘thieves’, I mean those who were not commercial sex workers. (Male clients FGD 4, person 2)

By mainly using CSWs as peer educators the intervention was unable to challenge the context of stigma around sex and low levels of communication about HIV and sexual health between couples.

### Message to ‘use condoms’ failed to engage with complex reality: strong resistance to condoms

Although the intervention strongly promoted the message that community members should stick to one partner, there was an acceptance that many people had casual partners, stressing the importance of promoting condom use. Peer educators promoted condom use in their educational meetings and had condom distribution targets (of 1000 per month). Boxes of free condoms were placed in clinics and public toilets throughout the intervention communities. However, STIs and HIV continued to be contracted at the same rates as in the control communities, suggesting that the intervention efforts failed to increase the use of condoms.

The FGDs and interviews suggest that the interventions message to “use condoms” did not meaningfully engage with the social realities of the target community. While some people commented that they learned how to actually put on and dispose of a condom, there was no evidence that the intervention had discussed widespread local resistance to condom use and strategies to increase condom use in the face of this resistance, as this quotation highlights: “They educated us on condom use but they never gave us practical information or assistance on behaviour change” (CSW 1, FGD site 7). People certainly heard and remembered the pro-condom message—many interviewees, including CSWs, parroted the idea that condoms should be used—but there was overwhelming evidence that most people did not like condoms and did not use them.

During the focus group discussions, reference was made to numerous complex reasons why the community resisted condom use. We now briefly outline the reasons why people did not use condoms and how they perceived the intervention as having failed to engage with this resistance in a meaningful way.

First, both men and women spoke frequently of enjoying sex more without condoms. Second, condoms were closely associated with use between a sex worker and client so community members felt it was very difficult to initiate the use of condoms between a husband and wife, which they believed would have been a useful programme outcome. Third, among CSWs, condoms were seen to indicate a high level of emotional and social distance; when a client and sex worker continued to have sex beyond two or three times condoms were expected by both parties to be discarded because the couple become ‘close’, even though it remained a commercial client-sex worker arrangement. Fourth, the blue condoms distributed free by the intervention were specifically disliked, especially in comparison to another brand of condoms (Protector Plus) available on the market. Reasons for this dislike stemmed from suspicions that the condoms were from European or American donors who infected them with HIV. In addition, people did not value the intervention condoms because they were sure that free things were of poorer quality, suggesting they were made of cheaper material than the Protector Plus condoms and irritated the skin and caused rashes. However, the Protector Plus condoms were cited as too expensive. Fifth, clients of CSWs were suspicious of female condoms because they thought CSWs reused them from client to client. Sixth, women in general were suspicious of male condoms because they thought some men would prick the condom to spread HIV or get women pregnant. An additional factor may have been that clients offered more money for condom-free sex, which was attractive to CSWs who were very poor. However, this factor was debated with some informants arguing that there was no difference in price.

Resistance to condoms was complex, multifaceted and deeply ingrained across many sections of society, particularly CSWs, their clients, and married couples. Failure to use condoms can be linked to perceived low vulnerability to HIV, a symbolic link between condom-less sex and intimacy, fatalistic attitudes about HIV and death, female distrust and suspicion of men, male distrust and suspicion of women (particularly CSWs), and many other social psychological community issues. It could be seen as overwhelming to attempt to break down the issues underlying such strong resistance. However, not engaging with these reasons for disliking condoms and promoting only a simplistic message that condoms should be used (which is how local people reported experiencing the intervention) was not effective. Local people did not feel that the intervention’s key message on condom use engaged or resonated with their local realities.

Reflecting the theoretical approach to ‘realistic evaluation’, our analysis points to a web of inter-related factors that, in the view of community members, served as obstacles to the success of the programme. This analysis not only underscores the importance of supplementing effectiveness evaluations with qualitative process evaluations in order to contextualise the outcomes and impacts observed, but also makes the case for conducting in-depth process evaluations throughout the project cycle (and not merely at the end of the project). Arguably, had a process evaluation of this kind been conducted earlier on in the project cycle, some of these challenges could have been identified, and changes made to the programme.

## Conclusion

The reasons for success or failure of any programme are likely to be multi-faceted. In this paper, we have sought to add to understandings of the outcome of the Manicaland STI/HIV Intervention through eliciting the views of target community members and local programme implementation staff on the peer education component.

Local programme implementers commented that their efforts were impeded by inconsistent buy-in and support from project partners in the NGO and public sectors. The perceived programme expectation that sex workers would reform their behaviour was undermined by the failure of its income generation efforts in a harsh economic climate. Given the lack of alternative survival strategies for women, programme pressures on sex workers to reform probably perpetuated the already strong stigmatisation of this group of women (both by themselves and by the wider community). In the light of the large academic literature on the negative impacts of stigma on prevention efforts, an approach which recognised and accepted the economic pressures on women to engage in sex work might have been more successful. Although the income generating projects constituted a form of recognition of such pressures, these were not viable at the time of the intervention due to increased inflation. The latter approach is standard practice with sex worker programmes in other parts of the world, and is frequently cited as one of the determining factors of success in sex worker projects in India, for example [25,26].

In addition, local people took issue with the use of sex workers as peer educators. Many people we spoke to regarded this as an unwise strategy for various reasons. Community members argued that the programme was undermined by its use of sex workers as bearers of pro-condom and pro-fidelity messages, since they are widely known to be sexually promiscuous and often condom averse. They said that the use of a highly stigmatised group (sex workers) and highly stigmatised venues (beer halls) as the major focus of peer education efforts distanced the programme from more ‘respectable’ community members.

Although the rationale of peer education involves the promotion of debate and dialogue amongst community members, with particular focus on obstacles to behaviour change and how these can be overcome, the community members felt the programme involved didactic messages on the importance of condom use. As a result, whilst the programme seems to have increased knowledge about condoms, it did not seem to have made any progress in dislodging pre-existing resistance to using them.

This paper highlights the ways in which local community networks have strong potential to undermine externally developed programs. New information and behaviour cannot be embraced by communities without engaging with local knowledge and resistances to change, and without taking account of local constraints on action.

## Endnotes

^a^The interviewers were affiliated with one of the NGOs that had implemented the intervention but were not personally involved.

^b^Zimbabwe suffered from very high inflation during the years of the intervention. The rates were 1999: 58%, 2000: 56%, 2001: 132%, 2002: 139%, 2003: 385% [27].
